# NATbox: a network analysis toolbox in R

**DOI:** 10.1186/1471-2105-10-S11-S14

**Published:** 2009-10-08

**Authors:** Shweta S Chavan, Michael A Bauer, Marco Scutari, Radhakrishnan Nagarajan

**Affiliations:** 1 UALR/UAMS Joint Bioinformatics Program, University of Arkansas at Little Rock, Arkansas, USA; 2 Department of Statistical Sciences, University of Padova, Italy; 3 Department of Biostatistics, University of Arkansas for Medical Science, USA

## Abstract

**Background:**

There has been recent interest in capturing the functional relationships (FRs) from high-throughput assays using suitable computational techniques. FRs elucidate the working of genes in concert as a system as opposed to independent entities hence may provide preliminary insights into biological pathways and signalling mechanisms. Bayesian structure learning (BSL) techniques and its extensions have been used successfully for modelling FRs from expression profiles. Such techniques are especially useful in discovering undocumented FRs, investigating non-canonical signalling mechanisms and cross-talk between pathways. The objective of the present study is to develop a graphical user interface (GUI), *NATbox: Network Analysis Toolbox *in the language R that houses a battery of BSL algorithms in conjunction with suitable statistical tools for modelling FRs in the form of acyclic networks from gene expression profiles and their subsequent analysis.

**Results:**

NATbox is a menu-driven open-source GUI implemented in the R statistical language for modelling and analysis of FRs from gene expression profiles. It provides options to (*i*) impute missing observations in the given data (*ii*) model FRs and network structure from gene expression profiles using a battery of BSL algorithms and identify robust dependencies using a bootstrap procedure, (*iii*) present the FRs in the form of acyclic graphs for visualization and investigate its topological properties using network analysis metrics, (*iv*) retrieve FRs of interest from published literature. Subsequently, use these FRs as structural priors in BSL (*v*) enhance scalability of BSL across high-dimensional data by parallelizing the bootstrap routines.

**Conclusion:**

NATbox provides a menu-driven GUI for modelling and analysis of FRs from gene expression profiles. By incorporating readily available functions from existing R-packages, it minimizes redundancy and improves reproducibility, transparency and sustainability, characteristic of open-source environments. NATbox is especially suited for interdisciplinary researchers and biologists with minimal programming experience and would like to use systems biology approaches without delving into the algorithmic aspects. The GUI provides appropriate parameter recommendations for the various menu options including default parameter choices for the user. NATbox can also prove to be a useful demonstration and teaching tool in graduate and undergraduate course in systems biology. It has been tested successfully under Windows and Linux operating systems. The source code along with installation instructions and accompanying tutorial can be found at http://bioinformatics.ualr.edu/natboxWiki/index.php/Main_Page.

## Background

Classical biological experiments have focused on understanding changes in the expression of single genes across distinct biological states. Such differential gene expression analyses while useful may not provide sufficient insight into their interactions or functional relationships (FRs). Understanding FRs is crucial as genes work in concert as a system as opposed to independent entities. On a related note, phenotype formation is mediated by pathways comprising of complex interactions between several genes as opposed to a single gene. Recent development of high-throughput assays in conjunction with sophisticated computational tools has enabled modelling such interactions and gain system-level understanding.

Several commercial and non-commercial software packages have been developed in the past for modelling gene interactions. Ontology-based packages [1,2] that rely on prior knowledge have been used traditionally to identify pathways enriched in a given experiment from existing documented pathways. Commercial packages (Ingenuity Pathway Analysis, Ingenuity Systems, Redwood City, CA) and (Pathway Studio, Ariadne Genomics, Rockville, MD) provide menu-driven GUI for retrieving functional relationships on a given set of genes from published literature. It is important to note that such techniques draw conclusions based on documented pathways and pooling knowledge across disparate sources. This in turn can render the conclusions noisy as genes and FRs may exhibit considerable variations across studies. Such an approach also relies implicitly on prior information, hence may have limited use in discovering novel FRs. Recent studies have provided compelling evidence of non-canonical signalling mechanism and cross-talk between pathways [3,4] that demand inferring network structure from the given data as opposed to direct inference from documented/curated pathways.

Bayesian structure learning (BSL) techniques [5] have been used successfully to infer interactions between a given set of genes in the form of graphs. The inherent probabilistic nature of gene expression and access to high-throughput assays that facilitate simultaneous measurement of transcriptional, translational and post-translational activities [4] are some of the reasons for their wide-spread use. Gene expression data with interventions [4] have also been recently shown to improve the conclusions drawn using Bayesian network modelling [4]. Probabilistic mechanisms underlying gene expression can be attributed to inherent noisiness and heterogeneity within/between cell population(s) [6]. High-throughput assays such as microarrays [7,8] and clonal gene expression profiling [9] in conjunction with BSL [10] had been used successfully in the past to capture functional relationships at the transcriptional level. More recently, high-throughput flow cytometry data from single-cells with perturbations in conjunction with BSL were used to obtain system-level understanding at translational and post-translational data [4].

**Figure 1:**
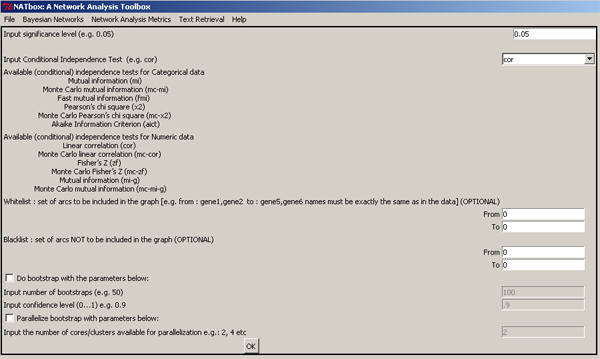
**Network Analysis Toolbox (NATbox)**. A screen shot of the Network Analysis Toolbox (NATbox) showing the main menu and the interface for Bayesian structure learning (BSL) with parameter recommendations and default values.

Several open-source packages are available for BSL and can be used to model gene networks [11-13]. However, these packages expect the user to be familiar with the programming environment and syntax. NATbox, Fig. 1, aims to overcome some of these obstacles by providing a menu-driven GUI for modelling and analysis of gene expression networks. It invokes functions from existing R-packages to accomplish this [14]. Reusing existing functionalities is strongly encouraged in an open-source environment and can minimize redundancy while improving transparency, reproducibility and sustainability. The target audience of NATbox are interdisciplinary researchers and biologists who wish to explore the tools of systems biology and network modelling for investigating signalling mechanisms. NATbox does not require the user to be familiar with R-programming or the algorithmic intricacies. Suitable parameter recommendations along with popular default parameter choices are provided as required to the user. As with any R-package, NATbox is open-source, hence lends itself to be customized by users with expertise in R. A detailed Wiki help page accompanies the toolbox and provides a systematic overview of the toolbox functions along with examples AT http://bioinformatics.ualr.edu/natboxWiki/index.php/Main_Page.

## Results

Prior to a detailed description of NATbox functionalities, we briefly review those of a closely related package BNArray [13] which was designed to provide a higher-level abstraction of R-routines from existing packages for gene network modelling. BNArray is a command line driven package where the user is expected to be familiar with the R environment [14] and syntax. It consists of four main modules (*i*) determine missing values in a given data using the functions *LLSimpute*, also implemented in the R-package *pcamethods *[15] (*ii*) Learn acyclic network structure using BSL routines from the R-package *deal *[16]. Subsequently display the graph by invoking functions from the R-package *dynamicGraph *[17] (*iii*) identify what the author term as 1^*st *^order network structure using a resampling procedure and (*iv*) reconstruct coherent sub-regulatory networks using an extended version of the algorithm CODENSE [18]. In the following discussion, we describe the features of NATbox as well as compare those implemented in BNArray in a systematic manner.

The comparisons are also enclosed under Table 1 for a quick review.

**Table 1:** Comparison of main features in NATbox and BNArray

NATbox	BNArray
Provides a Graphical User Interface (GUI)	Command-line driven with no graphical user interface (GUI).
Supported across operating systems: Windows and Linux.	Supported across operating systems: Windows and Linux.
Parameter recommendations are provided for the various algorithms with default values automatically inserted in GUI.	No parameter recommendations and default values are provided for the algorithms.
Input file is tab-delimited where columns represent the genes of interest and rows represent the experiments. The rows are assumed to be independent of one another.	Input file is tab-delimited where rows represent the genes of interest and columns their measurements across experiments. Unlike NATbox, no specifications are provided as to whether the experiments need to be correlated or uncorrelated.
Imputation of missing values is accomplished by k-nearest neighbour approach *impute.knn *from the R-package *impute*	Imputation of missing values is accomplished by local least square estimation *LLSImpute *also implemented in the R-package *pcamethods.*
Bayesian Structure Learning (BSL) algorithms are invoked from the R-Package *bnlearn.*	Bayesian Structure Learning (BSL) algorithms are invoked from the R-Package *deal.*
BSL algorithms ideally suited for *continuous as well as discrete data sets *characteristic of gene expression profiles and their quantized/coarse-grained counterparts.	BSL algorithm is ideally suited for *mixed data type *consisting of continuous as well as discrete values. Although, the authors of BNArray use it for analyzing temporally correlated gene expression data.
BSL algorithms from the package *bnlearn *include constraint-based (GS, IAMB, Fast-IAMB, IIAMB, MMPC) as well as search and score techniques (Hill-Climbing) are implemented.	No options are provided for multiple BSL algorithms.
BSL techniques implemented from *bnlearn *provide choice of several conditional independence tests and scoring criteria for continuous and discrete data sets under constraint-based and search and score techniques respectively. *Conditional independence tests for constraint-based*: mutual information, mutual information for Gaussian distributed data, fast mutual information, Pearson X^2^, Akaike information criterion, linear correlation and Fisher's Z. *Scoring Criteria for search and score*: multinomial likelihood, multinomial log-likelihood, Akaike information criterion, Bayesian information criterion, Bayesian Dirichlet score and Gaussian posterior density).	No options are provided for multiple BSL algorithms.
Provides options to incorporate structural priors during BSL by whitelisting (include) and blacklisting (exclude) edges.	No options are provided for incorporating structural priors.
Confidence of an edge is determined by bootstrapping. Uses R-package RGraphviz for visualization, which is designed to handle the layout of large graphs. Provides options to highlight edges whose confidence is greater than user specified threshold.	Confidence of an edge is determined by bootstrapping. Uses R-package *dynamicGraph *for visualization, which may require manual tuning of the node layout for large graphs. No options are provided to highlight edges whose confidence is greater than user specified threshold.
Parallelization of the bootstrap routines is accomplished by invoking functions from the R-package *SNOW*.	No options are provided for parallelization.
Topological properties of the results of BSL are investigated using centrality measures (degree, betweenness and closeness) from the *R-package igraph.*	Does not provide any centrality measures.
Provides motif finder from the package *igraph *for identifying motifs from the results of BSL. Results from *igraph *can also be loaded into *Cytoscape *fro detailed visualization.	Provides a modified version of the algorithm CODENSE for constructing coherent sub-networks from the results of BSL.
Provides a text retrieval interface to retrieve published literature to retrieve functional relationships of interest. This is useful justifying the choice structural priors in BSL.	No interface for text retrieval is provided.

*i. ***GUI**: NATbox provides a convenient menu-driven graphical user interface (GUI) developed using Tcl/Tk for modelling and analysis of gene expression networks. This has to be contrasted with BNArray [13], which is a command line driven package and demands the user to be familiar with the R-environment and syntax.

*ii. ***Input/output**: The input data in NATbox is assumed to be in tab-delimited text format, similar to that of BNArray. In NATbox, the rows of the input file represent *independent *experiments whereas the columns represent the names of the genes. However, in BNArray the rows represent the genes of interest and the columns represent their expression across experiments. Unlike NATbox, BNArray does not specify whether the experiments need to be independent or dependent.

*iii. ***Missing values**: Gene expression profiles often have missing values. For instance, in microarray data such missing values are common and may be attributed to experimental artefact, improper hybridization and non-specific binding of the probes. It is prudent to use suitable statistical techniques to accommodate such data sets rather than discard them. NATbox provides an option to determine missing values using nearest-neighbour averaging approach (**menu**: *File*), *impute.knn *(R-package *impute*) which has been found to perform well for high-dimensional data sets [19]. BNArray also provides a neighbourhood-based imputation technique, namely the least local squares (*LLSImpute*) [15] algorithm for determining missing values. A version of LLSImpute algorithm is also available through the (R-package *pcamethods*).

*iv. ***Functional relationships**: NATbox provides the option to model functional relationships using a battery of Bayesian structure learning techniques from the R-package (*bnlearn*) [20] (**menu**: *Bayesian Networks*). It is important to note that they model the network structure solely from the joint probability distribution in the absence of explicit temporal information. The tab-delimited input data should be in the form of a matrix where the columns represents the number of genes, and rows the number of repeated (independent) experiments. Each element in the matrix represents the expression value of that gene in a given experiment.

Constraint-based techniques in NATbox that model the network based on the results of conditional independence tests include:

• Grow-Shrink Algorithm (GS) [21]: GS is based on the Grow-Shrink Markov blanket, the first (and simplest) Markov blanket detection algorithm.

• Incremental Association Markov Blanket Algorithm (IAMB) [22]: IAMB is based on the Markov blanket detection algorithm. It uses a two-phase selection scheme. A forward selection followed by an attempt to remove false positives which scales well up to thousands of variables.

• Fast Incremental Association Markov Blanket Algorithm (Fast-IAMB) [23]: Fast-IAMB is a variant of IAMB which uses speculative stepwise forward selection to reduce the number of conditional independence tests.

• Interleaved Incremental Association Markov Blanket Algorithm (Inter-IAMB) [22]: Inter-IAMB is another variant of IAMB that uses forward stepwise selection to avoid false positives in the Markov blanket detection phase. Even though it often requires more conditional tests than the other IAMB variants, it's still able to scale up to thousands of variables while maintaining its robustness against false positives.

• Max-Min Parents Children Algorithm (MMPC) [24]: MMPC is a forward selection technique for neighbourhood detection based on the maximization of the minimum association measure. It learns the underlying structure of the Bayesian.

Several choices of conditional independence tests for categorical/discrete (*mutual information, mutual information for Gaussian distributed data, fast mutual information, Pearson's χ*^2^, *Akaike information criterion*) as well as numerical/continuous distributions (*linear correlation, Fisher's Z*) are provided. NATbox also provides an interface to the *search and score algorithm (HC, Hill-climbing*) from *bnlearn*. HC searches the model space and retrieves the best model using a scoring criterion which is also provided. Several choices of scoring criterion are provided for categorical/discrete (*multinomial likelihood, multinomial log-likelihood, Akaike information criterion, Bayesian information criterion, Bayesian Dirichlet score, K2 score*) and numerical/continuous distributions (*Gaussian posterior density*). Each BSL technique works under implicit assumptions and may result in spurious conclusions when these assumptions are violated. NATbox provides a battery of BSL techniques to alleviate such concerns. For instance, constraint-based techniques can be affected by sample sizes and are sensitive to initial results of the conditional independence tests. Search and score algorithms can result in local optima, hence may benefit from multiple random restarts unlike constraint-based approaches.

On the other hand, BNArray [13] uses a greedy search implementation of the BSL technique implemented in *deal *to learn the network structure. It is important to note that algorithm implemented in *deal *is ideally suited for handling *mixed data types *consisting of continuous as well as discrete data types. Although the authors [13] of BNArray have demonstrated the performance of their approach on temporal gene expression profile, it is unclear as to whether *deal *algorithm accommodates such temporal explicit information during structure learning. This is in contrast with NATbox which implements algorithms from *bnlearn *for learning the network structure from *homogenous *data types characteristic of gene expression profiles, i.e. either continuous or discrete data sets.

**Figure 2:**
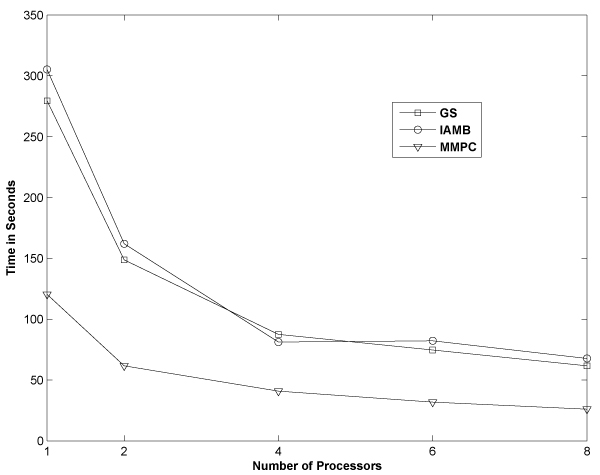
**Performance of BSL with bootstrap parallelization**. The performance of three BSL techniques (GS, IAMB and MMPC) with increasing number of processors (*np *= 1, 2, 4, 6 and 8) and 1000 bootstrap simulations, across an 8-processor Linux machine for the data [4]. Each of these algorithms exhibits (~5 fold) decrease in computational time at (*np *= 8) compared to (*np *= 1).

*v. ***Bootstrap parallelization**: Bootstrap techniques are commonly used for confidence estimation from a given empirical sample. Confidence [7] of an edge is determined by bootstrapping the given empirical data with replacement. Edges whose confidence is greater than a user-defined threshold (0 <*θ *< 1) are deemed robust. Bootstrap procedures can be computationally demanding for high-dimensional data sets. Search and score techniques such as hill-climbing implicitly require several random restarts during the confidence estimation. NATbox provides an option to parallelize bootstrapping across multiprocessor or multi-core processor by invoking the appropriate routines from the R-package *SNOW *(*Simple Network Of Workstations*) [25]. Such parallelization fall under embarrassingly parallel problems. However, they can significantly reduce the overall time complexity. Parallelization of the BSL routines themselves was not found to yield any significant improvement with increasing number of processors. We believe this may be due to inherent overhead in the master-worker configuration in SNOW. This is still under investigation. The performance for the various structure learning algorithms with increasing number of bootstraps and processors on BSL of the protein expression data [4] is shown in Fig. 2. A marked decrease in computational time is observed with increasing number of processors. The computational time with 1 processor is almost 5 folds of that obtained by 8 processors across the three algorithms (GS, IAMB and MMPC) and 1000 bootstrap simulations.

**Figure 3:**
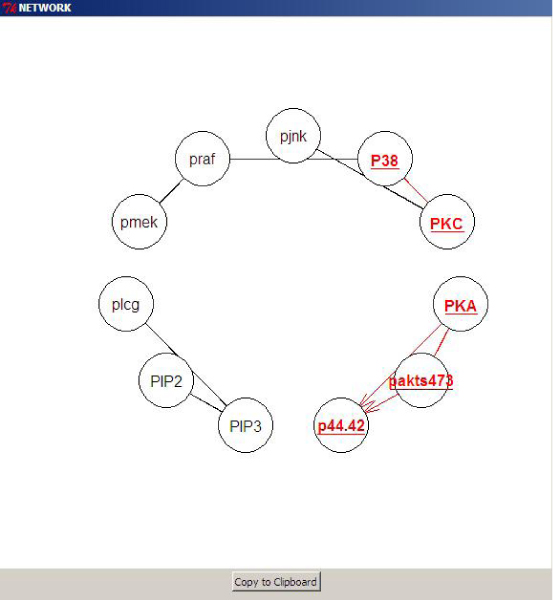
**Highlight Robust FRs**. The network structure learned using the Grow-Shrink (GS) algorithm from the data [4]. Robust FRs are deemed as those whose confidence is greater than the user-defined threshold (*θ *> 0.8), highlighted in red.

The results of the bootstrap are written onto a tab-delimited file. An option is also provided for highlighting the robust FRs (*θ *> 0.8) on the acyclic graph learned from the given data, Fig. 3. BNArray also implements bootstrap routines to determine what the author call as first-order network structures. Unlike NATbox, BNArray does not provide any options for bootstrap parallelization. BNArray uses routines from the package *dynamicGraph *[17] for visualization of the resulting acyclic network.

In addition, NATbox GUI also provides options for incorporating (directional) structural priors in BSL by *whitelisting *(include) and *blacklisting *(exclude) FRs. Such priors impose constraints on the network structure which implicitly rely on prior knowledge and needs to be chosen prudently in order to avoid bias during learning. However, a proper choice can alleviate uncertainty and improve accuracy of the conclusions. A text retrieval interface is provided for identifying structural priors and can be useful for investigating well-established signalling mechanisms.

*vi. ***Network analysis metrics**: BSL techniques are useful in inferring the cause-effect relationships and network structure from the gene expression profiles. However, they provide no insight into the network's topological properties. NATbox incorporates social network analysis metrics and motif finder from the package (*igraph*) [26] under (**menu**: *Network Analysis Metrics*) for investigating the topological properties of the networks generated using BSL techniques. Such metrics can be especially useful in investigating large networks. The input is assumed to be a binary adjacency matrix of the network constructed using BSL with ones and zeros representing the presence/absence of an edge respectively. Since BSL results in directed acyclic graphs, the corresponding binary adjacency matrix need not be symmetric. The centrality measures (*degree centrality, closeness centrality, betweenness centrality, alpha centrality*) along with their respective parameter options are provided for the user. A detailed discussion of these centrality measures are deferred to [26,27]. Degree centrality is one of the commonly used measures of centrality and broadly classified into out-degree (edges directed outward from a node) and in-degree (edges directed into a node). The distribution of the degree centrality has been widely studied and provides crucial insight into the topology of a network. Recent studies have elucidated the prevalence of power-law degree distribution in biological networks [28]. Such power-law distributions may be accompanied by interesting properties such as the presence of hubs (critical genes) and scale-free phenomena unlike exponential degree distributions. Justification of power-law degree distributions can be challenging especially when the information about the tail of the distribution is insufficient [29]. Betweenness centrality is useful in identifying genes that act as important mediators between other genes, although they themselves might not be densely connected to all the packages. Genes with high betweenness centrality may play an important role in bridging the gap between densely connected subgroups. On the other hand, closeness centrality is useful in identifying genes that are directly or indirectly dependent on other genes. The menu also provides an option to determine the diameter and identify recurrent structures or motifs of sizes 3 and 4 in the given network by invoking the respective functions from *igraph *[26]. Motifs are recurrent atomic structures with frequency distribution different from that of random network. Recent studies have identified certain specific motifs that persist across distinct real world networks [30]. The *igraph *package incorporated into NATBox also provides option to save the acyclic network in formats compatible with Cytoscape [31] for detailed visualization.

**Figure 4:**
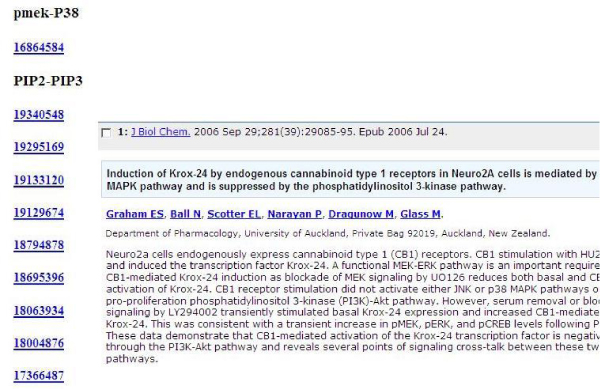
**Text retrieval interface for structural priors**. Results of text retrieval interface (html file) revealing prior literature (PubMed identifiers) on one of the robust FRs (PIP2-PIP3) identified by BSL of the expression data [4]. These results in turn can be used to impose structural priors, hence refine BSL.

*vii. ***Retrieving FRs from literature**: Finally, NATbox provides options for retrieving FRs of interest using NCBI *ESearch *(**menu**: *Text Retrieval*). The user has the option to input the (*a*) pairs of gene names (co-occurrence) of interest through the GUI or (*b*) upload a two-column matrix of FRs of interest or those identified as robust by the bootstrap procedure. For well-documented studies, an integrated approach that incorporates the results from the *Text Retrieval *(structural prior) in justifying the choice of *whitelisted *(include) and/or *blacklisted *(edges) in BSL. The *Text Retrieval *results are in html format, with a list of PUBMED identifiers hyper-linking to the respective abstracts/articles in PUBMED. The results of text retrieval on gene expression data from [4] is shown in Fig. 4.

## Conclusion

Modelling and analysis of gene expression networks is an area of active research. Several tools have been proposed in the literature for the same. Recently, Bayesian structure learning (BSL) techniques in conjunction with high throughput assays were used successfully to capture functional relationships. Existing packages may demand the user to have considerable programming expertise. NATbox provides a convenient menu-driven GUI along with appropriate parameter recommendations including default parameter choices for modelling and analysis of gene expression networks. It incorporates diverse functionalities from existing R-packages. This in turn encourages transparency and reproducibility, characteristic of open-source environment. NATbox can also be used as a teaching and demonstration tool for graduate courses in systems biology. Immediate future enhancements to the toolbox include (*i*) expand the choice of structure learning algorithms including dynamic bayesian networks (*ii*) improve statistical inference of the network features (*ii*) parallelization of the implemented routines across multi-core and multi-processor machines of BSL functions as well as bootstrapping (*iii*) provide a web-interface so as to obviate the need for local installation of the toolbox (*iv*) enhance text retrieval so as to accommodate advanced text mining approaches.

## Competing interests

The authors declare that they have no competing interests.

## Authors' contributions

SSC and MAB implemented the toolbox under RN's guidance. MS developed the *bnlearn *package and was involved in trouble shooting the Bayesian structure learning routines. RN wrote the manuscript.
